# Co-Expression of *JcNAC1*- and *JcZFP8*-Improved Agronomic Traits of Tobacco and Enhanced Drought Resistance through *NbbHLH1* and *NbbHLH2*

**DOI:** 10.3390/plants12173029

**Published:** 2023-08-23

**Authors:** Xianfei Niu, Zhiping Lai, Linghui Wang, Rui Ma, Yingying Ren, Xueying Wang, Cheng Cheng, Ting Wang, Fang Chen, Ying Xu

**Affiliations:** 1 Key Laboratory of Bio-Resources and Eco-Environment of Ministry of Education, College of Life Sciences, Sichuan University, Chengdu 610065, China; 2 College of Life Science and Food Engineering, Yibin University, Yibin 644000, China

**Keywords:** *JcNAC1*, *JcZFP8*, double gene co-expression, growth traits, drought stress

## Abstract

Previous studies have identified numerous transcription factors involved in drought response, each of which play different roles in plants. The objective of the present study was to evaluate the effectiveness of two transcription factors on drought response in *Jatropha curcas* L., *JcNAC1* and *JcZFP8*. The overexpression of these transcription factors in tobacco (*Nicotiana benthamiana* L.) improved drought resistance, but *JcZFP8* delayed germination and *JcNAC1* reduced biomass and yield. By constitutively co-expressing these two genes in tobacco, drought resistance was improved, and the negative effects of each of them were overcome. The transgenic plants with double-gene co-expression showed stronger drought tolerance with 1.76-fold greater accumulation of proline and lower H_2_O_2_ and malondialdehyde (MDA) content to 43 and 65% of wildtype (WT) levels, respectively. The expression levels of *NbbHLH1* and *NbbHLH2* genes upregulated linearly with the increased drought tolerance of double genes co-expression plants. In drought conditions, the leaf water contents of *bhlh1*, *bhlh2*, and *bhlh1bhlh2* deletion mutants obtained by CRISPR-CAS9 knockout technique were maintained at 99%, 97%, and 97% of WT. The *bhlh1bhlh2* was found with lower germination rate but with higher reactive oxygen levels (1.64-fold H_2_O_2_ and 1.41-fold MDA levels). Thus, the co-expression of two transcription factors with different functions overcame the adverse traits brought by a single gene and enhanced the shared drought-tolerant traits, which can provide guidance on theory and selection of gene combinations for the application of multi-gene co-expression in agriculture in the future.

## 1. Introduction

Drought stress affects plant survival and growth. To explore this adverse effect, scientists seek to breed highly drought-tolerant crops by transgenic technology or genetic modification methods [1,2]. However, different drought tolerance genes have different effects on plant drought resistance, and the strategy for using them in response to drought stress must take these differences into account. Drought tolerance genes are often associated with unfavorable plant growth traits, such as growth inhibition [3,4,5].

NAM/ATAF/CUC protein (NAC) and C2H2 zinc finger protein (ZFP) are involved in plant growth and stress responses [6,7]. *VaNAC17* improves drought tolerance in Arabidopsis by upregulating genes involved in JA signaling pathways and enhancing ROS scavenging [8]. *OsZFP15* reduces sensitivity to ABA and improves drought tolerance in rice [9]. *OsDRZ1* enhances the drought resistance of rice by enhancing antioxidant capacity [10]. The basic helix–loop–helix (bHLH) is involved in plant drought responses [11]. *NbbHLH1* and *NbbHLH2* function as positive regulators in the jasmonate signal transduction pathway [12]. *AhbHLH112* enhances ROS-scavenging ability by regulating Peroxidase (POD)-mediated H_2_O_2_ homeostasis [13]. *PtrbHLH66* improves the drought tolerance in Arabidopsis by increasing the proline contents and antioxidant enzyme activities, and reducing reactive oxygen species (ROS) and malondialdehyde (MDA) under drought stress [14].

Drought is a polygenic trait, and potential candidate genes contribute to cell detoxification, osmotic accumulation, antioxidant mechanisms, and signaling pathways [15]. The overexpression of both *OsPIL1* and *AtDREB1A* in Arabidopsis improves drought tolerance and plant growth [16]. The co-expression of *AtGA5* and *AtDREB1A* increases Arabidopsis biomass and flower induction, and leads to high levels of drought stress tolerance [17]. The co-expression of *NHX1* and *eIF4A1* from Arabidopsis positively regulates drought stress tolerance in sweet potato [18]. However, single gene transformation cannot meet our needs for drought tolerance and agronomic traits, and the co-expression of multiple genes is currently a major challenge in biogenetic engineering. Co-expressing stress response genes and growth regulatory genes can compensate for the accumulation of trade-off genes. This strategy can be effective in reducing adverse growth traits by the overexpression of stress response genes, but the mechanism of this strategy is extremely complex and less research has been conducted in this area [15,19].

Multiple research projects on drought resistance of transcription factors have mainly focused on a single transcription factor, while few studies have thoroughly investigated on the regulation of plant growth traits and drought resistance by co-expression of double transcription factors. *JcNAC1* and *JcZFP8* are two different transcription factors cloned from *Jatropha curcas* L. in our previous studies [20,21]. Experimental approaches transferred *JcNAC1* (NAC) and *JcZFP8* (ZFP) into *Nicotiana tabacum* L., and the results confirmed that plants with double gene co-expression (NZ) had preferable growth traits and drought tolerance. Using qRT-PCR and CRISPR-Cas9, we found that *JcNAC1* and *JcZFP8* enhanced the expression of *NbbHLH1* and *NbbHLH2*, and the knocking out tobacco of *NbbHLH1* and *NbbHLH2* reduces tolerance to drought. This combined pattern of transcription factors has potential applications in crop improvement programs.

## 2. Results

### 2.1. Co-Expression of JcNAC1/JcZFP8 Not Only Improves PEG-Drought Resistance, but Also Overcomes the Adverse Agronomic Traits Brought by Single Gene Transformation

#### 2.1.1. Co-Expression of *JcNAC1* and *JcZFP8* Can Increase the Germination Rate of Plants under Mannitol-Drought

**Figure 1:**
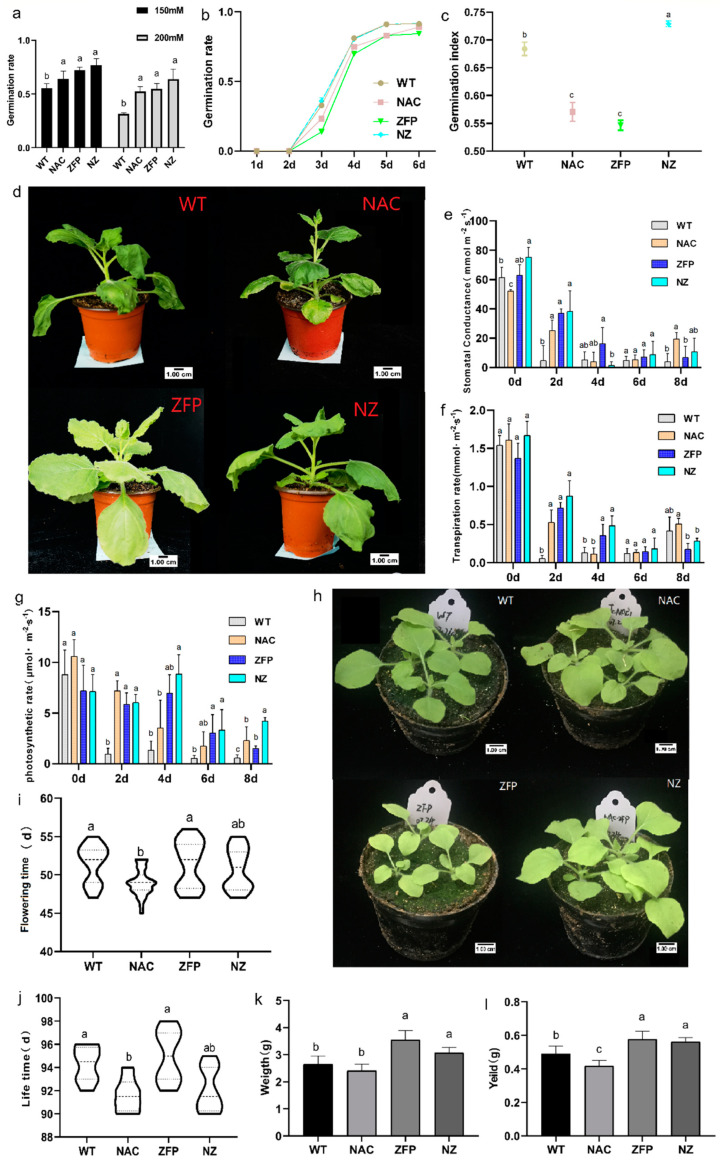
Growth and leaf gas exchange traits of WT and overexpression lines. (**a**) Germination rate of seeds treated with different mannitol concentrations; (**b**) germination rate in 1/2 MS medium; (**c**) germination index in 1/2 MS medium; (**d**) plant appearance after four days of 10%PEG6000 treatment (panels **e**–**g**); (**e**) stomatal conductance (mmol m^−2^·s^−1^); (**f**) transpiration rate (mmol m^−2^·s^−1^); (**g**) photosynthetic rate (μmol·m^−2^·s^−1^); (**h**) growth traits after three weeks; (**i**) flowering time; (**j**) life cycle time; (**k**) dry weight at harvest time; (**l**) seed yield at harvest time. Different letters indicate significant difference determined by one-way ANOVA with Tukey’s test (*p* < 0.05), data are means ± SEM.

We sought to determine the germination rate under the stress of low water potential (physiological drought), which we imposed using a high concentration of the osmoticum mannitol. When the mannitol concentration was 150 mM and 200 mM, the relative germination rates of NAC, ZFP, and NZ overexpression lines were significantly higher than WT (Figure 1a and Table S1). Increasing the mannitol concentration from 150 to 200 mM decreased the germination rate about 44% in WT, but only decreased it by about 17% in the overexpression lines. This suggests that the tolerance of NAC, ZFP, and NZ to mannitol-drought was higher than that of WT.

To understand the seed germination ability of transgenic plants, the number of germinated seeds was counted daily from the beginning of inoculation (Figure 1b). All lines started to germinate on day 3. Among them, the double overexpression line NZ had the fastest germination rate, followed by WT, NAC, and ZFP. According to the calculation of the germination index of the seeds of each strain, NZ had the highest germination index, and the germination index of NAC and ZFP were lower than WT (Figure 1c and Table S2), which indicated that the overexpression of *JcNAC1* and *JcZFP8* delayed seed germination. The results with the two-gene overexpression line NZ suggested that there was an interaction between two genes that enhanced seed germination rate.

#### 2.1.2. NAC, ZFP, and NZ Transgenic Tobacco Performed Better in PEG-Drought (Low Water Potential) Compared to WT

Transgenic lines improved seed germination under osmotic stress. Five-week-old tobacco treated with 10% PEG6000 for 4 days showed significant differences between WT and transgenic tobacco. WT showed obvious wilting, whereas the leaves of the overexpression lines of tobacco showed only slight wilting (Figure 1d).

Stomata are the structures that regulate plant transpiration. Under stress, the stomata of WT closed on day 2, and remained closed through day 8, while the stomata of NAC, ZFP, and NZ plants closed more slowly and had significantly higher stomatal conductance on day 2 than WT (Figure 1e and Table S3). Correspondingly, transpiration rate of WT decreased fastest and transpiration rate of NAC, ZFP, and NZ decreased more slowly (Figure 1f and Table S3).

The photosynthetic rate of WT decreased to near zero at day 2 of PEG osmotic stress whereas the overexpression lines maintained high photosynthetic rates at day 2, and the ZFP and NZ overexpression lines maintained high photosynthetic rates at day 4 (Figure 1g and Table S3). ZFP and NZ had higher photosynthetic rates than WT at day 6 and day 8 of stress as well.

#### 2.1.3. Double Gene Co-Expressing Plants Overcome the Adverse Agronomic Traits Brought by Single Gene Transformation

For the changes in germination of transgenic lines, we studied the growth traits of transgenic lines. At three weeks after planting, ZFP lines had only developed about 3.5 expanded leaves, whereas WT, NAC, and NZ lines had developed 4.5 leaves. Upon observing the leaf size, we found that ZFP had smaller leaves. Fewer and smaller leaves indicated ZFP’s early stage growth inhibition (Figure 1h and Figure S1).

Upon observing the flowering time of different plant lines, we found that the average flowering time from early to late was NAC, NZ, WT, and ZFP. The flowering period of NAC was 3 days earlier than WT (Figure 1i and Table S2). The average life cycle of NAC was 4 days shorter than that of WT and ZFP (Figure 1j and Table S2). However, in NZ, while flowering was intermediate between wildtype, ZFP, and NAC, the duration of the life cycle was short, as in NAC.

After harvesting the plants that had completed the life cycle, the biomass and yield of the different plants were statistically analyzed. The results showed that the average biomass of NZ and ZFP was significantly higher than WT and NAC, and the biomass of NZ was 16% higher than WT (Figure 1k and Table S2). The seed yields of NZ and ZFP were also significantly higher than WT, while the seed yield of NAC was lower than WT and the yield of NZ was 14% higher than WT (Figure 1l and Table S2). In conclusion, NZ obtained higher biomass accumulation and seed yield, suggesting that co-over-expression of the two genes in tobacco NZ may have overcome the germination delay and growth inhibition phenotype in early stage caused by the overexpression of *JcZFP8*, and the reduction in biomass and yield caused by the overexpression of *JcNAC1*.

### 2.2. More Proline and Less Reactive Oxygen Species (ROS) Level Endows the Double Gene Overexpression Lines with Stronger PEG-Drought Resistance

#### 2.2.1. Under 10%PEG6000 Treatment, the Accumulation of ROS in Transgenic Plants Was Less

**Figure 2:**
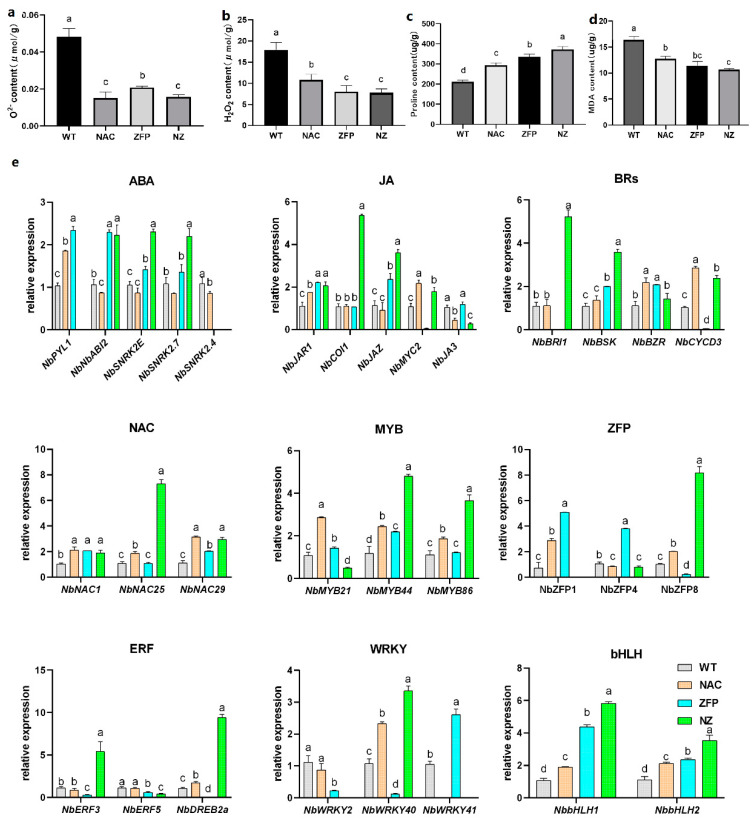
The effect of single-gene overexpression (NAC and ZFP) versus double gene co-expression (NZ) on tolerance to 10% PEG6000 osmotic stress treatment. (**a**) O^2−^ content; (**b**) H_2_O_2_ content; (**c**) Proline content; (**d**) MDA content; (**e**) transcription factor regulation involved in tobacco drought response. The superscript letters in the table represent the differences between different genotypes, and different letters indicate significant differences (*p* < 0.05), data are means ± SEM.

In this study, five-week-old plants were treated with PEG6000 at 10% concentration, to compare the ROS levels of WT and transgenic plants after 4 days of stress. NBT and DAB staining were used to provide a qualitative measure of the levels of O^2−^ and H_2_O_2_ (Figure S2a,b). According to the degree of leaf staining, it appeared that ROS accumulation in NAC, ZFP, and NZ was less than in WT. A quantitative assay of O^2−^ and H_2_O_2_ levels indicated that WT accumulated considerably higher concentrations of ROS than the overexpression lines; the accumulated O^2−^ of ZFP was higher than that of NAC and NZ (Figure 2a and Table S4), and the H_2_O_2_ content of NAC was higher than that of ZFP and NZ (Figure 2b and Table S4). The content of O^2-^ and H_2_O_2_ in NZ was 33% and 43% of that in WT.

Proline concentration was lowest in WT, and was considerably higher in the overexpression lines (Figure 2c and Table S4). The accumulation of proline in NZ plants was 1.76 times in WT and higher than that in NAC and ZFP.

At under 10% PEG6000 stress, the MDA content in WT was the highest, and was significantly lower in the other lines (Figure 2d and Table S4). The MDA accumulation of NZ plants was 65% of that of WT; the oxidative damage in NZ and ZFP was less than that in WT and NAC.

In conclusion, under drought stress, the co-expressed tobacco NZ had more proline and less ROS, which likely contributed to its tolerance of osmotic stress.

#### 2.2.2. *NbbHLH1* and *NbbHLH2* Genes Increased Linearly from WT to Single-Overexpression Lines to NZ under PEG-Drought

To further test the relationship between the transcription factors *JcNAC1* and *JcZFP8* in hormone signaling, we analyzed the expression of several key genes in the ABA, JA, and BR signaling pathways and the regulation of downstream transcription factors by qRT-PCR (Figure 2e). The expression of several of these genes was substantially higher in NZ than in WT and the other lines, while the single-gene overexpressed lines tended to have only slightly increased or the same level of expression as the WT. Those in which NZ was substantially higher than the other lines included: the ABA-related genes *NbSNRK2E* and *NbSNRK2.7*, the JA-related genes *NbCOI1* and *NbJAZ*, the BR-related genes *NbBRI1* and *NbBSK*, the NAC gene *NbNAC25*, the MYB-related genes *NbMYB44* and *NbMYB86*, the ZFP gene *NbZFP8*, the ERF-related genes *NbERF3* and *NbDREB2a*, the WRKY gene *NbWRKY40*, and the bHLH genes *NbbHLH1* and *NbbHLH2* (Figure 2g). Overall, the expression of other transcription factors was changed, but there was no obvious rule. The expression levels of *NbbHLH1* and *NbbHLH2* genes increased linearly from WT to single-overexpression lines to NZ.

### 2.3. The NbbHLH1 and NbbHLH2 Were Involved in Drought Resistance of Tobacco

#### 2.3.1. The Germination Rate of *bhlh1*, *bhlh2*, and *bhlh1bhlh2* Decreased under Mannitol Treatment

To further explore the effect of *NbbHLH1* and *NbbHLH2* on the drought-resistant characteristics of tobacco, we selected WT for gene knockout verification The CRISPR knockout technique was used to study the role of *NbbHLH1*, *NbbHLH2* in drought tolerance. The vectors *PHSE401-bHLH1A1B1*, *PHSE401-bHLH2A2B2*, and *PHSE401-bHLH1B2-bHLH2B2* were constructed. The mutants of *bhlh1*, *bhlh2*, and *bhlh1bhlh2* were obtained (Figures S4 and S5).

**Figure 3:**
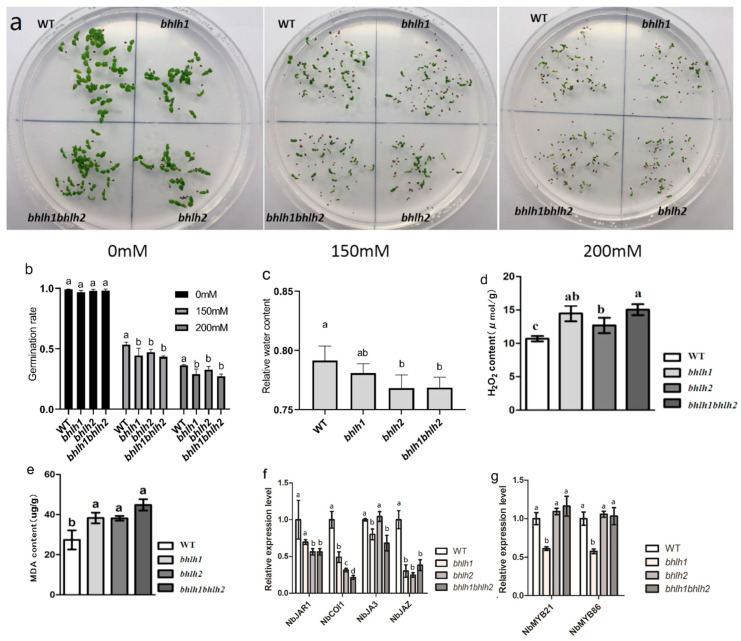
The effect of *bhlh1*, *bhlh2*, and *bhlh1bhlh2* knock-out on properties related to drought response. Properties in panels a and b were in plants subjected to mannitol osmotic stress; properties in panels c to g were measured on leaves of pot-grown plants that were subjected to drought by withholding water for 7 days. (**a**) germination and growth of *bhlh1*, *bhlh2*, and *bhlh1bhlh2* in control and mannitol medium; (**b**) germination rate of *bhlh1*, *bhlh2*, and *bhlh1bhlh2* under mannitol treatment; (**c**) relative water content of *bhlh1*, *bhlh2*, and *bhlh1bhlh2*; (**d**) H_2_O_2_ content; (**e**) MDA content; (**f**) expression of genes in the JA signaling pathway; and (**g**) gene expression of MYB transcription factors. The superscript letters in the table represent the differences between different genotypes, and different letters indicate significant differences (*p* < 0.05), data are means ± SEM.

The germination rate of the three knockout lines in 150 mM and 200 mM mannitol medium was substantially lower than in 0 mM mannitol medium, but in 150 and 200 mM mannitol, the knockout lines were only slightly lower, though significantly (*p* ≤ 0.05) than the WT (Figure 3a,b and Table S5).

The knockout lines at five weeks of age were stressed with withholding watering for 7 days. The relative water contents of *bhlh2* and *bhlh1bhlh2* leaves were significantly lower than in the WT, although the magnitude of the effect was relatively small (Figure 3c and Table S6). The relative water content of in *bhlh1*, *bhlh2*, and *bhlh1bhlh2* was 99%, 97%, and 97% of that in WT, respectively.

#### 2.3.2. H_2_O_2_ and MDA Was Increased in *bhlh1*, *bhlh2*, and *bhlh1bhlh2*

The knockout lines at five weeks of age were stressed with withholding watering for 7 days, MDA and H_2_O_2_ contents of mutant tobacco were higher than WT, indicating that *bhlh1*, *bhlh2*, and *bhlh1bhlh2* accumulate more ROS. (Figure 3d,e and Table S6). By comparing single knockout tobacco with double knockout tobacco, it was found that the H_2_O_2_ content of *bhlh2* was lower than that of *bhlh1* and *bhlh1bhlh2*, while the contents of MDA were higher than WT in all three mutants. The contents of MDA and H_2_O_2_ in the leaves of *bhlh1bhlh2* were 1.41 and 1.64 times that of WT, indicating that the ROS scavenging ability of *bhlh1bhlh2* tobacco was weaker than that of WT.

#### 2.3.3. *NbbHLH1* and *NbbHLH2* May Not Affect Drought Resistance by Regulating the Expression of *NbMYB21* and *NbMYB86*

Since *NbbHLH1* and *NbbHLH2* are important response genes downstream of JA signal transduction, we determined the regulation of the expression of key JA signaling genes in WT and mutant tobacco under drought conditions (Figure 3f). All three knockout lines had a lower expression than WT of *NbJAR1*, *NbCOI1*, and *NbJAZ*, but the expression of *NbJA3* was only slightly lower in *bhlh1* and *bhlh1bhlh2* than in WT. We analyzed the expression of MYB-type transcription factors in the knockout lines and found that two genes, *NbMYB21* and *NbMYB86*, were downregulated only in *bhlh1* mutants (Figure 3g). There was no significant difference in expression in the other two knockout lines compared with the WT, suggesting that *NbbHLH1* and *NbbHLH2* might not regulate the drought response by affecting the expression of *NbMYB21* and *NbMYB86*.

In conclusion, knock-out tobacco *bhlh1*, *bhlh2*, and *bhlh1bhlh2* had lower germination rate under mannitol, more MDA and H_2_O_2_ under drought, which indicates its weakened tolerance to drought stress.

## 3. Discussion

Drought stress is the most catastrophic stress affecting crops that has a serious impact on crop yield [22]. Most of the research on the application of transcription factors to drought resistance has focused on the effect of individual transcription factors on the resistance of plants [23]. The overexpression of a transcription factor alone can enhance drought resistance while often associating adverse growth traits such as retarded growth [5]. There are few reports of co-expression of different transcription factors to produce better growth and drought resistance traits in plants. We characterized *JcNAC1* and *JcZFP8* co-expression and found that it can overcome the unfavorable growth traits of single gene plants and engender stronger drought resistance. *JcNAC1* and *JcZFP8* synergistically enhanced the expression of *NbbHLH1* and *NbbHLH2* to improve the drought resistance of plants. This gene combination had a good balance between growth traits and stress resistance, which has great potential in agriculture.

Studies have shown that stress-induced promoters are crucial for achieving ideal expression of transcription factors, which can eliminate the damage of constitutive expression on plant growth and development [24]. By using the *Oshox24* promoter, *AtDREB1A* can enhance rice drought resistance through stress-induced expression while relieving the growth inhibition caused by constitutive expression [25]. *Prd29A*:*TaDREB2B* transgenic sugarcane can enhance drought resistance without affecting growth [26]. The inducible composition of *pAsr11875*:*SaADF2* in Arabidopsis enhances drought resistance while relieving the growth inhibition caused by constitutive expression [24]. In our study, both *JcNAC1* and *JcZFP8* were constitutively expressed and not only enhanced tobacco drought-resistance but also affected plant growth. The use of drought-induced promoters to achieve the induced expression of *JcNAC1* and *JcZFP8,* which can improving plant drought resistance without affecting plant growth, was considered. The strategy can be considered but requires further research.

The current studies showed that single-gene overexpression of *JcNAC1* or *JcZFP8* in tobacco decreased germination index, but the co-expression of these genes in NZ stimulated germination index to levels higher than WT (Figure 1c). In *Arabidopsis*, gibberellin (GA) can mediate endosperm expansion and regulate seed germination, and RGL2 in the GA pathway repressed activation of the *EXPA2* promoter by NAC25/NAC1L, while NAC1L has been identified as an upstream regulator of *EXPA2* expression [27]. *JcZFP8* affects tobacco plant height via GA [21]. According to these studies, the co-expression of *JcNAC1* and *JcZFP8* may play a role in the GA/DELLA-NAC-EXPA2 network, to promote seed germination. However, how these two genes function through this pathway requires further research.

*OsERF83* improves drought tolerance in rice but also causes growth inhibition and reduced yield [28]. *AmDREB1F* increases the drought tolerance of Arabidopsis, but constitutive expression also leads to a phenotype of delayed growth [29]. *OsTZF5* improves the survival rate of rice under drought stress and leads to growth inhibition [3]. The constitutive expression of *GmNAC085* in *Arabidopsis* improves drought tolerance and also results in delayed growth of aboveground and root [30]. These transcription factors all enhance plant drought resistance while causing plant growth inhibition. *JcZFP8* enhanced tobacco drought resistance and also caused delayed germination and early growth inhibition. We also used qRT-PCR to determine the expression of some NAC, ZFP, and ERF transcription factors in NAC, ZFP, and the co-overexpression line (Figure 2e). While ZFP overexpression improved tolerance to osmotic stress (Figure 1k,l), ZFP alone did not significantly upregulate the tested stress-related transcription factors (Figure 2e). Zinc finger proteins in Arabidopsis are involved in enhancing stress tolerance, but often have a negative effect on growth [10]. In *ZFPL*-overexpressing plants, the expression of photosynthesis-related genes was downregulated, resulting in plant growth inhibition [31]. In the current study, the overexpression of *JcZFP8* transiently maintained photosynthetic rate on day 2 after drought stress treatment (Figure 1g). In previous studies, *JcZFP8* affected the growth of *Nicotiana tabacum* L. via GA, resulting in plant dwarfing [21]. It is possible that other types of transcription factors are regulated by *JcZFP8*, resulting in ZFP improving plant drought resistance and inhibiting growth.

ZFP exhibited growth inhibition at three weeks of age, but there was no significant difference between the double gene co-expression line NZ and WT (Figure 1h and Figure S1). The NAC protein is located at the branching point of the ABA-dependent and independent pathway, which can avoid cross talk between stress resistance genes and growth genes [32]. The interaction between the GA inhibitor DELLA and NAC protein in cotton mediates GA signal transduction [33]. *ScNAC23* can accelerate GA-mediated flowering and senescence in Arabidopsis [34]. *JcZFP8* inhibits plant growth and development via GA, whereas *JcNAC1* can regulate plant flowering and premature senescence by enhancing GA signaling. *JcNAC1* and *JcZFP8* may work antagonistically via GA to regulate the growth of NZ, avoiding early flowering and senescence caused by *JcNAC1*, accelerating growth inhibition caused by *JcZFP8* and ensuring the agronomic advantage of NZ.

NAC, ZFP, and NZ accumulated less reactive oxygen species and more proline under osmotic stress, endowing plants with stronger osmotic stress tolerance (Figure 1d–g and Figure 2a–d). Under drought stress, *CarNAC4* in Arabidopsis resulted in lower MDA and low water loss [35]. *SlNAC10* improved drought tolerance by increasing proline synthesis [36]. The gene expression in jasmonic acid (JA) synthesis and the signaling of *VaNAC17*-expressing Arabidopsis plants is upregulated under drought stress, resulting in the reduced accumulation of reactive oxygen species in plants [8]. The overexpression of *ZF2* in Arabidopsis enhanced drought tolerance by increasing proline [37]. The overexpression of *OsDRZ1* in rice improves plant drought resistance by reducing ROS, and increasing proline [10]. These studies suggest that NAC and C2H2-ZFP transcription factors can regulate drought resistance by reducing ROS accumulation, increasing proline content, and regulating the expression of downstream jasmonic acid response genes. We found that NAC, ZFP, and NZ improved resistance to drought stress in these ways. The content of O^2−^ and H_2_O_2_ in NZ was lower, but NZ had significantly increased proline content, which ensured stronger tolerance to drought stress in NZ.

Our results demonstrated that the drought resistance of the double-gene transformed lines was stronger than that of the single-gene transformed lines, and the drought resistance was superimposed in the double-gene transformed lines. *PpNAC2* and *PpNAC3* were related to the JA response in pine [38]. AtZP1 regulates genes encoding *bHLH* transcription factors [39]. *NbbHLH1* and *NbbHLH2* were upregulated in transgenic plants; in addition, superimposed expression was found in the double gene co-expressed plants. These results indicate that these two transcription factors are involved in the increasing drought resistance of transgenic plants.

Under drought stress, the mutant tobacco *bhlh1*, *bhlh2*, and *bhlh1bhlh2* accumulated more H_2_O_2_ and MDA (Figure 3d,e). *MYC2* was involved in plant drought tolerance associated with JA [40]. *PxbHLH02* increased the drought tolerance of poplar with lower H_2_O_2_ [41]. *MdCIB1*-transgenic Arabidopsis exhibited drought tolerance with lower MDA and H_2_O_2_ accumulation [42]. *NbbHLH1* and *NbbHLH2* were positive regulators in the JA pathway [11]. The JAZ-MYC module was a central component of JA response [43]. Our studies provide evidence that *NbbHLH1* and *NbbHLH2* may not regulate the expression of *NbMYB21* and *NbMYB86*, Further research is needed on how *NbbHLH1* and *NbbHLH2* regulate tobacco drought resistance through the JA pathway.

## 4. Materials and Methods

### 4.1. Construction and Transformation of JcNAC1, JcZFP8, and JcNAC1-JcZFP8

*JcNAC1* and *JcZFP8* were cloned from the cDNA of *Jatropha curcas* L. [21,22]. *pBI121* vector was digested with *BamHI* and *SacI*, the *JcZFP8* gene was then cloned into the *pBI121* (named *pBI121*-*JcZFP8*) vector. *pCMBIA1302* vector was digested with *BamHI* and *HindIII*, the *JcNAC1* gene was then cloned into the *pCMBIA1302* (named *pCMBIA1302*-*JcNAC1*) vector. *JcZFP8* and *JcNAC1* were driven by the CaMV35S promoter (Figure S3a).

*pBWA(V)B2C* vector was digested with *BsmBI*, the *JcNAC1* gene was then cloned into the *pBWA(V)B2C* (named *pBWA(V)B2C*-*JcNAC1*) vector by ClonExpress ultra one step cloning Kit (Vazyme). *pBWA(V)B2C*-*JcNAC1* vector was digested with *Esp3I*, the *JcZFP8* gene was then cloned into the *pBWA(V)B2C*-*JcNAC1* (named *p*BWA(V)B2C-*JcNAC1-JcZFP8*) vector by ClonExpress ultra one step cloning Kit (Vazyme) (Figure S3a). The *pBWA(V)B2C* vector was purchased from BioRun Bio (Wuhan, China) and had two *Ubi* promoters that can overexpress two different genes simultaneously.

After identification and sequencing, the correct recombinant vectors were transformed into *Agrobacterium* GV3101, which was used for genetic transformation as previously described [44]. Positive transgenic plants were selected as follows: Single gene overexpression *JcNAC1* lines (named NAC) by hygromycin (50 mg/L), Single gene overexpression *JcZFP8* lines (named ZFP) by kanamycin (50 mg/L) and the double-gene overexpression *JcNAC1-JcZFP8* lines (named NZ) by basta (20 mg/L). The expression of *JcNAC1* and *JcZFP8* in T_1_ were verified by PCR and qRT-PCR (Figure S1b). Subsequent experiments were performed using T_2_ tobacco.

All primers were synthesized by Sangon (China), the primer sequences are shown in (Table S7).

### 4.2. Construction and Transformation of NbbHLH1, NbbHLH2, and NbbHLH1–NbbHLH2

The sequences of *NbbHLH1 (NbS00019773g0102)* and *NbbHLH2 (NbS00001919g0002)* were extracted from previous studies [12]. sgRNA for *NbbHLH1* and *NbbHLH2* were designed on the website https://crispr.dbcls.jp/ (last access date 13 August 2023), and different knockout vectors of *NbbHLH1* and *NbbHLH2* were constructed on PHSE401 [45]. The vector was transformed into *Agrobacterium* GV3101 and then used for genetic transformation as described in 2.1 (Figure S5). Knocked-out lines of *NbbHLH1* (named *bhlh1*) and knocked-out lines of *NbbHLH2* (named *bhlh2*) were selected by hygromycin (50 mg/L). The knockout results in T_1_ were validated by PCR and sequencing (Figure S4a,b). We selected T_2_ *bhlh1* and *bhlh2*, which can only be knocked out at one site, for subsequent experiments.

Based on the knockout results of *bhlh1* and *bhlh2*, we selected sgRNA for *NbbHLH1* and *NbbHLH2* to construct the knockout vector (Figure S5) and obtained double knocked-out lines (named *bhlh1bhlh2*) using the method described above. Double knocked-out lines of *bhlh1bhlh2* were selected by hygromycin (50 mg/L). We selected T_2_ *bhlh1bhlh2* for subsequent experiments.

### 4.3. Plant Materials and Drought Stress Treatments

*Nicotiana benthamiana* L. was used in this study. Seeds were sterilized with 75% alcohol for 3 min and planted on 1/2 Murashige Skoog (MS) medium (1/2 MS medium containing 3% sucrose and 0.7% agar). Seven days later, tobacco was planted in the pot (Diameter × Bottom diameter × Height: 90 mm × 60 mm × 75 mm) full of soil (nutrient soil: vermiculite = 1:1) at 25 °C with a light density of ~120 μmol m^−2^ s^−1^ under a photoperiod of 16/8 h in greenhouse.

WT and transgenic tobacco “*JcNAC1*(NAC), *JcZFP8* (ZFP) and *JcNAC1-JcZFP8*(NZ)” at five weeks of age were stressed with 10% PEG6000 for 4 days. WT and knockout tobacco “*bhlh1*, *bhlh2* and *bhlh1bhlh2*” at five weeks of age were stressed with withholding watering for 7 days. The second fully unfolded leaf from the top to the bottom was selected, frozen in liquid nitrogen for 30 min, and stored in a −80 °C low-temperature refrigerator for use.

### 4.4. Germination Rates and Other Growth Trait

Sterilized seeds of WT, overexpression lines (NAC, ZFP and NZ), and knockout lines (*bhlh1*, *bhlh2*, and *bhlh1bhlh2*) were placed on 1/2 MS medium with mannitol (0 mM, 150 mM, 200 mM). The seed germination rates were counted and assayed with three biological replicates. Germination index = $\sum \frac{n_{i}}{t_{i}}$, *n_i_* is the number of germinated seeds on the *i*th day (*t_i_*) in percentage by days^−1^ [46].

### 4.5. Flowering Period, Life Cycle, Weight, and Yield

Flowering time was defined as full expansion of the first flower. Life cycle was defined as the first upper leaf turning completely yellow. After the plant was fully harvested, the above-ground parts were oven-dried at 70 °C for 3 days and the seeds were oven-dried at 37 °C for 7 days and weighted. Twenty-four biological replicates of each sample were collected for analysis [47].

### 4.6. Photosynthesis, Stomatal Conductance, and Transpiration Rate

Five-week-old WT, NAC ZFP, and NZ leaves were treated with 10% (*w*/*v*) PEG 6000, and stomatal conductance, transpiration rates, and photosynthesis were measured with GFS-3000 (WALZ-Cor, Effeltrich, Germany). Data were collected at 0, 2, 4, 6, 8 days of 10% PEG 6000 treatment [48]. Nine biological replicates were prepared for each plant group.

### 4.7. Expression Analysis of Important Genes in Drought Treatments

Plant leaves were stored in −80 °C low-temperature refrigerator for use in Section 2.3. Total RNA was extracted using Kit from Foregene (Chengdu, China). The cDNA synthesized and qPCR performed using Kit from Vazyme (Nanjing, China), all primers were synthesized by Sangon (Shanghai, China), the primer sequences are shown in (Table S1), and the reaction system and procedure were performed according to Shi et al. [21]. Three biological replicates were prepared for each plant group.

### 4.8. Measurements of Physiological Parameters

Fresh leaves of WT, NAC, ZFP, and NZ lines were collected after 10% PEG6000 treatments for 4 days for measurement of O^2−^ and H_2_O_2_, malondialdehyde (MDA), and proline level using the related test kits (Solarbio, Beijing, China); we followed the instructions of the reagent kit for specific operations. [49].

Fresh leaves of WT, *bhlh1*, *bhlh2*, and *bhlh1bhlh2* knockout lines at five weeks of age were stressed with withholding watering for 7 days for measurement of physiological parameters related to drought, including relative water content (RWC), MDA, and H_2_O_2_ [50].

The second fully unfolded leaf from the top to the bottom was selected with three biological replicates.

RWC = (FW − DW)/(TW − DW) × 100% (FW: fresh weight; DW: dry weight; TW: turgid fresh weight).

## 5. Conclusions

In this study, the co-expression of two different transcription factors *JcNAC1* and *JcZFP8* in tobacco demonstrated better growth and drought resistance traits. Under 10% PEG6000 treatment, transgenic plants, especially the double overexpression line NZ, showed better drought tolerance, accumulating more proline and less H_2_O_2_ and less MDA. In transgenic plants. *NbbHLH1* and *NbbHLH2* manifested a superimposed effect, which played significant roles in the increased drought resistance of transgenic plants. Under drought treatment, the drought resistance of the knock-out lines *bhlh1*, *bhlh2*, and *bhlh1bhlh2* was weakened, and more H_2_O_2_ and MDA were accumulated. We provided evidence that *NbbHLH1* and *NbbHLH2* might not regulate the drought response by affecting the expression of *NbMYB21* and *NbMYB86* in the JA pathway. Our findings can provide guidance on theory and selection of gene combinations for application of multi-gene co-expression in agriculture in the future.

## Data Availability

The data supporting this study’s findings are available from the corresponding author upon reasonable request.

## Supplementary Materials

The following supporting information can be downloaded at: https://www.mdpi.com/article/10.3390/plants12173029/s1, Figure: S1 Growth traits of three weeks of WT and overexpression lines; Figure S2: ROS of single-gene overexpression (NAC and ZFP) versus double gene co-expression (NZ) on tolerance to 10% PEG6000 osmotic stress treatment; Figure S3: Construction of overexpressed plants and Map of genes overexpression vector; Figure S4: Sequence analysis of *NbbHLH1* and *NbbHLH2* gene knockout tobacco; Figure S5: Schematic diagram of double target vector construction; Table S1: Germination rate of overexpressed tobacco under Mannitol germination stress.; Table S2: Growth traits of WT and overexpression lines 4; Table S3: Leaf gas exchange traits of WT and overexpression lines under PEG-drought; Table S4: Effect of single-gene overexpression (NAC and ZFP) versus double gene co-expression (NZ) on tolerance to 10% PEG6000 osmotic stress treatment.; Table S5: Germination rate of knock-out tobacco under Mannitol germination stress; Table S6: Effect of knock-out tobacco on tolerance drought stress; Table S7: Primers used in this study.
