# Supplementary material for: Co-Expression of JcNAC1- and JcZFP8-Improved Agronomic Traits of Tobacco and Enhanced Drought Resistance through NbbHLH1 and NbbHLH2
Source: Plants (Basel). 2023 Aug 23;12(17):3029. doi: 10.3390/plants12173029 (PMC10490288; doi:10.3390/plants12173029)
Supplement: Supplementary file 1 [file plants-12-03029-s001.zip › plants-2485679-supplementary.pdf]

Supplementary materials

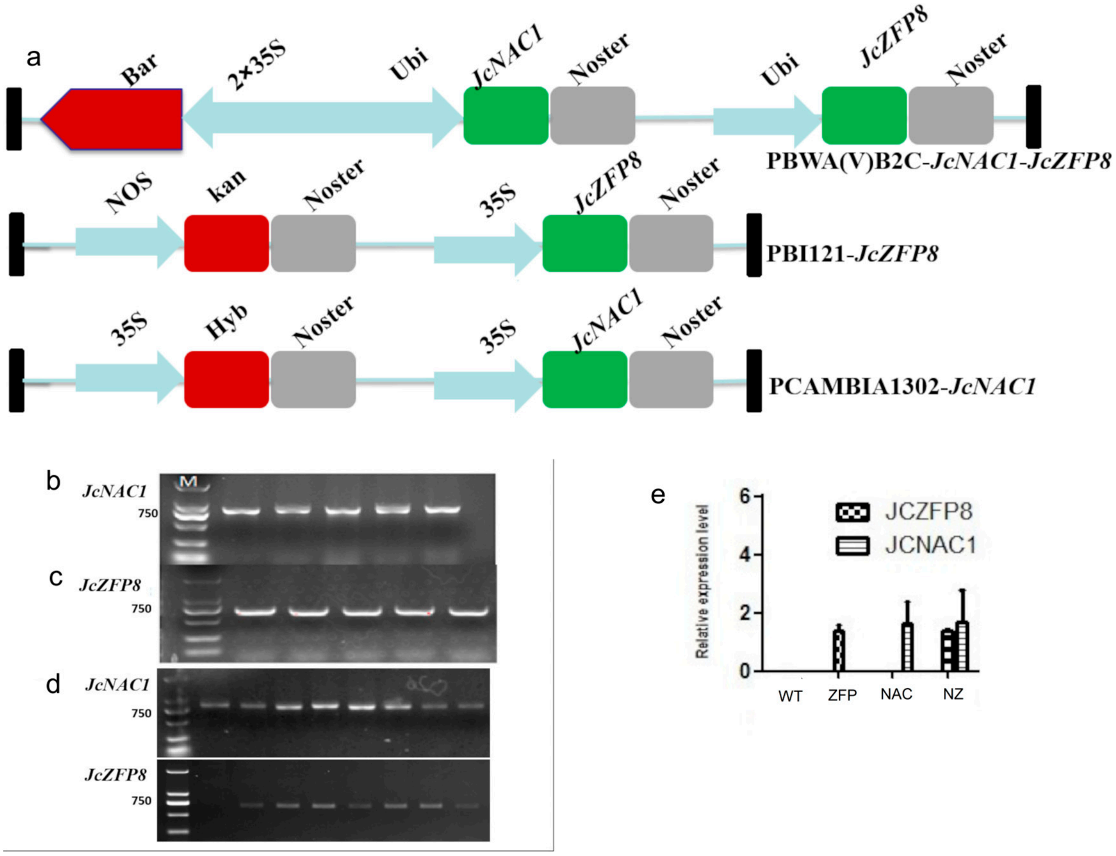

Figure S1 Construction of overexpressed plants a Map of genes overexpression vector; b Verification analysis of PCAM1302-***JcNAC1*** transgenic tobacco; c verification analysis of PBI121-***JcZFP8*** transgenic tobacco; d verification analysis of ***JcNAC1-JcZFP8*** double transgenic tobacco; e Genes expression level detection of transgenic tobacco M: 2000 Maker, other electrophoresis lanes represent different independent transformations lines, values are means ±SEM.

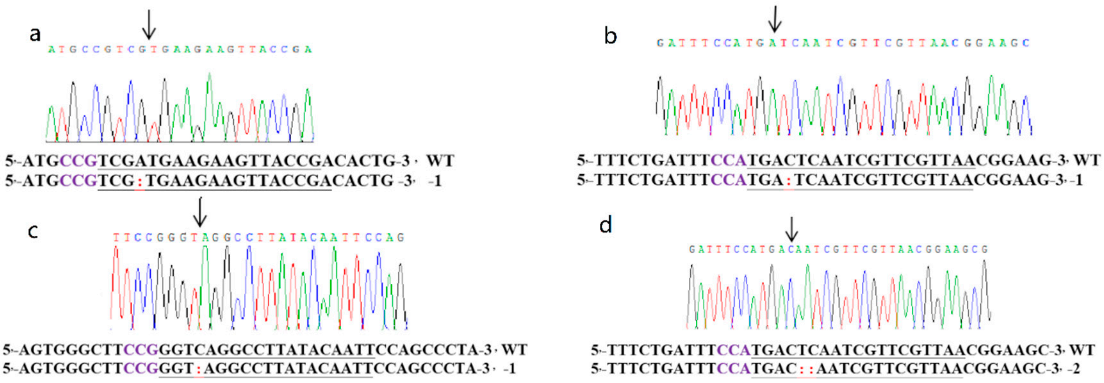

Figure S2 Sequence analysis of ***NbbHLH1*** and ***NbbHLH2*** gene knockout tobacco

a ***bhlh1***; b ***bhlh2;*** c and d ***bhlh1bhlh2*** ,The "–" indicating deletion of the given number of nucleotides. Deleted bases are marked with colon

Table S1 Primers used in this study

Construction of vector	*JcZFP8-F*	CGGGATCCATGGATAAGAGCGAAAGAG
	JcZFP8-R	CGAGCTCCTAAACTCACATGGTCTTG
	JcNAC1-F	CGGGATCCATGGCAGCAGCGGCGGCGT
	JcNAC1-R	CCCAAGCTTTCAGAACGGCTTCTGTAGG
qRT-PCR	*JcZFP8-F*	ATGGATAAGAGCGAAAGAG
	JcZFP8-R	CAGATGAAGATCTAAACTCACA
	JcNAC1-F	ATGGCAGCAGCGGCGGCGTTAG
	JcNAC1-R	GAACGGCTTCTGTAGGTACA
	NbPYL1-F	CAGGGTTGACACCAGAA
	NbPYL1-R	TGTGCGAGTAGGGAAGA
	NbNbABI2-F	CCCACTATCCAAGCACC
	NbNbABI2-R	TTGTACCACGAGCCAGA
	NbSNRK2E-F	TTGCCACCGAGACTTGA
	NbSNRK2E-R	CTGCGAATGTAACACTGAGGA
	NbSNRK2.7-F	GATTTGGGTTCTGGTAA
	NbSNRK2.7-R	CAGTGGACTTGGGTTGA
	NbSNRK2.4-F	GATTTGGGTTCTGGTAA
	NbSNRK2.4-R	CAGTGGACTTGGGTTGA
	NbJAR1-F	GGTACGGCTTGATTCCTG
	NbJAR1-R	TACTCCAACCCATCCTTC
	NbCOI1-F	TGTGCTATACGGCGAAAC
	NbCOI1-R	CCGAATCCCTAACAATCA
	NbJAZ-F	TCTGAGCCTTCAACACCA
	NbJAZ-R	GACGCCGTTTCTTCTTCT
	NbMYC2-F	CTCAGAGGCAGCACAATC
	NbMYC2-R	CAGCACCTGTAATCCAAA
	NbJA3-F	TCGGTGACGGGAACTG
	NbJA3-R	CACGACGGACGATTGC
	NbBRI1-F	AACCACCAGTTCCTACCT
	NbBRI1-R	TTTGGCGGATTCTTTAC
	NbBSK-F	TGGACGATGAGGTTGAG
	NbBSK-R	TCTGGAGGAGTGAAAGC
	NbBZR-F	TATCGCAAGGGAAACAGG
	NbBZR-R	TATGGAGAAAGGGCAGGA
	NbCYCD3-F	ATCGCAAGGGAAACAGG
	NbCYCD3-R	ATGGAGAAAGGGCAGGA
	NbNAC1-F	ATCGCAAGGGAAACAGG
	NbNAC1-R	ATGGAGAAAGGGCAGGA
	NbNAC29-F	GCTGCTGTTTCAGGTTAT
	NbNAC29-R	GAGCCACTTTGCTTGTTA
	NbNAC25-F	TCCTCTTCCCGTTGCTAT

	NbNAC25-R	TCCTATCCCTTGGACTGA
	NbMYB21-F	TGGATGCCAAGGTTAGTC
	NbMYB21-R	GGTAGCGGCTGTTGATTT
	NbMYB86-F	TCCGCCTCTTAGGTTGT
	NbMYB86-R	GGATTCATTGCCGAGTG
	NbMYB44-F	TCAACGGAGTGATGTAGC
	NbMYB44-R	CTGGTGAGGCACTAATCT
	NbZFP8-F	TCAACGGAGTGATGTAGC
	NbZFP8--R	CTGGTGAGGCACTAATCT
	NbZFP4-F	TGCCGAGGTGATGGAACA
	NbZFP4-R	CCCAAAGCCTGCGAACTA
	NbZFP1-F	CCGTGAGAAAGGTAATG
	NbZFP1-R	GTCCTCCTAATGCTTGT
	NbERF3-F	GACCCGTTTATTGACCC
	NbERF3-R	AACAACAGAAGACGAGGAG
	NbERF5-F	GAGAAGTGGAGGGTGAAA
	NbERF5-R	AATGGTGACAAAGGTGGC
	NbDREB2a-F	CAAAGTGGTGGTGCTAT
	NbDREB2a-R	GATGCCTTAGATGTGGA
	NbABF-F	TCGGATTATGGAGTTGCT
	NbABF-R	AGACTGTCGTTGTGGGTG
	NbWRKY40-F	TTTGCTCCTACCTGCCTCG
	NbWRKY40-R	TTGGGTTCTGTGCTGTTGA
	NbWRKY2-F	GCTGGATTTCACCCTAA
	NbWRKY2-R	TTGCTGATAAACCGATG
	NbWRKY41-F	TGCTCCTTATTCCTCCTC
	NbWRKY41-R	TGGCGATTCAGACCTTCC
	NbSbHLH1-F	CATGACCCCATCGTTTGTTAAC
	NbSbHLH1-R	TTGTCTGAAGCCCGAATCC
	NbSbHLH2-F	TGCCGTAGACCCATTAACATC
	NbSbHLH2-R	CCCCGTCGATTAAAGTCTGAAG
Construction of vector	*NbbHLH1*A1-BsF	ATATATGGTCTCGATTGTCGGTAACTTCTTCATCGAGTT
	*NbbHLH1*A1-F0	TGTCGGTAACTTCTTCATCGAGTTTTAGAGCTAGAAATAGC
	*NbbHLH1*B1-R0	AACGGTCAGGCCTTATACAATTCAATCTCTTAGTCGACTCTAC
	*NbbHLH1*B1-BsR	ATTATTGGTCTCGAAACGGTCAGGCCTTATACAATTCAA
	*NbbHLH2*A2-BsF	ATATATGGTCTCGATTGTTAACGAACGATTGAGTCAGTT
	*NbbHLH2*A2-F0	TGTTAACGAACGATTGAGTCAGTTTTAGAGCTAGAAATAGC

	*NbbHLH2*B2-R0	AACGAAATTAAACAATTCAGCTCAATCTC TTAGTCGACTCTAC
	*NbbHLH2B*2-BsR	ATTATTGGTCTCGAAACGAAATTAAACAA TTCAGCTCAA
	*NbbHLH2*A2-BsF	ATATATGGTCTCGATTGTTAACGAACGATT GAGTCAGTT
	*NbbHLH2*A2-F0	TGTTAACGAACGATTGAGTCAGTTTTAGA GCTAGAAATAGC
	*NbbHLH1*B1-R0	AACGGTCAGGCCTTATACAATTCAATCTC TTAGTCGACTCTAC
	*NbbHLH1*B1-BsR	ATTATTGGTCTCGAAACGGTCAGGCCTTA TACAATTCAA
